# FeCl_3_催化/铁源双功能制备咔唑基超交联磁性聚合物及其对水体中痕量四环素类抗生素的富集与检测

**DOI:** 10.3724/SP.J.1123.2026.01037

**Published:** 2026-11-08

**Authors:** Qingshan ZHU, Yuwei LIU, Tongtong QIN, Li MA, Wenjie ZHAO

**Affiliations:** 1. 河南工业大学化学化工学院，河南 郑州 450001; 1. School of Chemistry and Chemical Engineering，Henan University of Technology，Zhengzhou 450001，China; 2. 吉林省药品检验研究院，吉林 长春 130028; 2. Jilin Institute for Drug Control，Changchun 130028，China

**Keywords:** 磁固相萃取, 咔唑, 超交联聚合物, 四环素类抗生素, 环境水样, magnetic solid-phase extraction （MSPE）, carbazole, hypercrosslinked polymer, tetracycline antibiotics, environmental water samples

## Abstract

本研究以富电子、结构刚性的咔唑为功能单体，甲醛缩二甲醇（FDA）为交联剂，三氯化铁（FeCl_3_）为催化剂兼铁源，制备了新型磁性咔唑基超交联聚合物Fe_3_O_4_/car-HCP，经系统表征，阐明了其化学结构、形貌、孔道性质与磁性特征。该材料集成了咔唑芳香骨架对TCs的强*π-π*堆积与氢键作用、超交联网络的高疏水性以及Fe_3_O_4_的快速磁响应性，实现了对目标物的多机制协同吸附。将其作为磁性固相萃取（MSPE）吸附剂，结合高效液相色谱-紫外检测（HPLC-UV），建立了同时检测环境水样中土霉素（OTC）、四环素（TC）和强力霉素（DC）的分析方法。通过系统优化，确定最佳萃取条件为吸附剂用量20 mg、萃取时间30 min、萃取温度30 ℃、洗脱剂为乙腈-0.01 mol/L NaOH（体积比为2∶1）、洗脱时间20 min、洗脱体积3 mL。在优化条件下，方法表现出良好的分析性能：OTC、TC和DC的线性范围分别为2.83~200 μg/L、2.77~200 μg/L和9.93~200 μg/L，检出限（LOD，*S/N*=3）分别为0.83、0.80和2.98 μg/L；定量限（LOQ，*S/N*=10）分别为2.83、2.77和9.93 μg/L。将方法应用于实际水样（自来水、雨水及景观湖水）分析，在10 μg/L和100 μg/L两个加标水平下，目标物的平均回收率为75.6%~128.5%，相对标准偏差（RSD）为1.3%~9.4%，表明方法准确可靠、基质适用性强。与文献方法相比，本研究制备的新材料在保证高灵敏度与准确度的同时，显著减少了吸附剂用量并简化了操作流程。基于该材料优异的吸附性能和便捷的磁分离特性，本研究建立的分析方法成功实现了环境水样中痕量四环素类抗生素的灵敏检测，证实了其在复杂基质分析中的应用潜力。此外，所提出的“一物两用”合成策略也为设计多功能磁性吸附材料提供了新的思路。

## Abstract (en)

This study prepared a novel magnetic carbazole-based hypercrosslinked polymer （Fe_3_O_4_/car-HCP）. Electron-rich and rigid carbazole served as the functional monomer. Formaldehyde dimethyl acetal （FDA） was used as the crosslinker. Ferric chloride （FeCl_3_） acted as both catalyst and iron source. Systematic characterization revealed the material’s chemical structure，morphology，pore properties，and magnetic behaviour. Specifically，the material exhibited a high specific surface area and a well-developed porous network. It also showed superparamagnetic properties with sufficient saturation magnetization for rapid magnetic separation. The material offers several advantageous features for analytical extraction. Its carbazole aromatic skeleton provides strong *π*-*π* stacking interactions with the aromatic rings of tetracycline antibiotics （TCs）. The same skeleton also supplies hydrogen bonding sites，primarily through its nitrogen atom. Both types of interactions exhibit high affinity toward TCs. The hypercrosslinked network confers high hydrophobicity to the material. This property effectively minimizes non-specific adsorption of polar matrix components commonly present in environmental water samples，such as humic acid and inorganic salts. The embedded Fe_3_O_4_ nanoparticles enable rapid magnetic responsiveness. This allows convenient and fast separation of the adsorbent from the sample solution using an external magnet，eliminating the need for centrifugation or filtration steps. These structural and physicochemical features work together in a synergistic manner. Consequently，the material achieves multi-mechanistic adsorption of the target analytes，leading to high extraction efficiency. Using this material as a magnetic solid-phase extraction （MSPE） adsorbent，an analytical method was developed. The MSPE procedure was coupled with high performance liquid chromatography-ultraviolet detection （HPLC-UV）. The method allows for the simultaneous determination of three TCs in environmental water samples. The three TCs are oxytetracycline （OTC），tetracycline （TC），and doxycycline （DC）. Several extraction parameters were systematically optimized to achieve the best performance. The parameters included adsorbent dosage，extraction time，extraction temperature，eluent composition，elution time，and elution volume. The optimal extraction conditions were as follows： The sample was extracted with 20 mg of adsorbent at 30 ℃ for 30 min，and then eluted with 3 mL of acetonitrile/0.01 mol/L NaOH （2∶1，volume ratio） for 20 min. Under these optimized conditions，the method demonstrated satisfactory analytical performance. The linear range was 2.83–200 μg/L for OTC，2.77–200 μg/L for TC，and 9.93–200 μg/L for DC. The limits of detection （LODs），based on signal-to-noise ratio （*S*/*N*）=3，ranged from 0.83 μg/L to 2.98 μg/L. The limits of quantification （LOQs，*S*/*N*=10） ranged from 2.77 μg/L to 9.93 μg/L. The method was then applied to the analysis of real water samples. Three types of water were tested： tap water，rainwater，and landscape lake water. Recovery tests were conducted at two spiked concentration levels，namely 10 μg/L and 100 μg/L. The average recoveries of the three target analytes ranged from 75.6% to 128.5%. The relative standard deviations，calculated from three replicate measurements，were between 1.3% and 9.4%. These results confirm that the method is accurate，reliable，and applicable to complex aqueous matrices. Compared with previously reported methods for TC analysis，the present approach offers several distinct advantages. It requires significantly less adsorbent. It also simplifies the operational procedure by eliminating time-consuming steps. Importantly，these improvements are achieved without sacrificing sensitivity or accuracy. In summary，this study provides a robust，convenient，and efficient analytical solution for monitoring trace levels of TCs in environmental water. Furthermore，the design and synthesis strategy of this multifunctional magnetic adsorbent offers new insights for developing advanced functional materials for environmental sample preparation.

四环素类抗生素（tetracyclines，TCs）是以四并苯为母核的广谱抑菌药物，常见土霉素（oxytetracycline，OTC）、四环素（tetracycline，TC）和强力霉素（doxycycline，DC）。因成本低、抗菌谱广，TCs被广泛应用于临床治疗、畜牧养殖及促生长剂添加^［1，2］^；然而，该类药物在生物体内代谢不完全，残留物易进入水环境，不仅可能诱导抗生素耐药基因（如*tetK*、*tetM*）的传播，威胁生态安全^［3］^，还可经食物链累积于人体，引发胃肠、肝肾损伤，并对儿童骨骼与牙齿发育造成不可逆危害^［4］^。因此，建立快速、灵敏的环境水体中痕量TCs的检测方法，对环境污染控制与风险评估具有重要意义。

环境水样基质复杂且目标物浓度低，直接分析难度较大，高效的前处理技术成为关键^［5］^。磁性固相萃取（MSPE）因操作简便、分离快速、溶剂用量少、易于固液分离等优势，逐渐成为替代传统固相萃取的有力手段^［6］^。近年来，研究者开发了多种功能化磁性纳米复合材料用于TCs的富集，这些材料通过引入特异性作用位点或识别结构来提升吸附选择性。例如，生物质衍生的磁性多孔碳或羟基化碳纳米管等磁性碳材料可通过*π-π*相互作用及络合作用高效吸附TCs，并表现出吸附‑催化协同降解的潜力^［7］^。磁性金属有机骨架（MOFs）/共价有机框架（COFs）材料（如Fe_3_O_4_@MIL-101（Cr）-NH_2_）具有高比表面积和可设计功能位点，能通过多重作用实现TCs富集，已用于复杂食品基质检测^［8］^。为提升选择性，基于模板法的磁性分子印迹聚合物（如以TC为模板的Fe_3_O_4_@MIP）可构建与目标物结构互补的印迹空穴，实现对TCs的特异性识别与分离^［9］^。然而，现有材料在实际环境水样中的应用仍受限：磁性碳与MOFs材料多依赖非特异性吸附，易受共存物干扰；部分MOFs材料存在水稳定性与酸碱耐受性不足的问题；分子印迹聚合物则常面临传质阻力大、印迹位点利用率低等挑战。因此，开发兼具高选择性、优异稳定性、快速传质动力学及良好环境适应性的新型磁性吸附剂，是提高TCs痕量分析效能的关键方向。

超交联聚合物（HCPs）是一类由C、H、O、N等轻元素通过稳定共价键构建的聚合物，具有合成方法灵活、比表面积高、稳定性好及易于功能化等优点，可作为理想的吸附材料^［10］^。将其与磁性纳米粒子复合，既能保持高吸附性能，又可实现磁分离，已广泛用于环境和食品中痕量有机污染物、兽药、农药、添加剂等的检测^［11，12］^。值得一提的是，利用三氯化铁（FeCl_3_）同时作为催化剂和磁性粒子的铁源，即“一物两用”策略，有效实现了磁性复合材料的绿色制备。例如，Hu等^［13］^以1-萘硼酸为单体、甲醛缩二甲醇（FDA）为交联剂，通过FeCl_3_催化的傅克烷基化反应构建硼酸功能化HCP骨架，而FeCl_3_又通过原位共沉淀与Fe^2+^反应生成Fe_3_O_4_纳米粒子，所得Fe_3_O_4_@HCP-BA可用于尿液中儿茶酚胺和羟基多环芳烃的同步萃取与检测。针对食品中邻苯二甲酸酯（PAEs）的迁移污染问题，Qin等^［14］^以苄基化*β*-环糊精为功能单体、FDA为交联剂，通过FeCl_3_“一锅法”制备了Fe_3_O_4_/BnCD-HCPP，利用环糊精的主客体包合作用与HCP的疏水作用协同吸附PAEs，方法检出限低至0.1 μg/L，在茶饮料样品中回收率为81.5%~118.5%，循环使用9次后吸附效率无显著下降。此外，Ma等^［15］^利用FeCl_3_催化含羧基单体与FDA聚合，通过共沉淀形成Fe_3_O_4_磁性纳米颗粒，实现了多环芳烃（PAHs）与季铵盐农药（QUATs）的分类萃取。该“一物两用”策略简化了操作步骤并减少了试剂消耗，同时保持了材料的高吸附性能与可功能化特点，为复杂基质中痕量污染物的高效分离提供了新思路。

在众多可用于构筑HCPs的单体中，咔唑因其富电子、刚性的芳香杂环结构备受关注，咔唑基多孔聚合物已在气体吸附、光催化及生物医学等领域展现出应用潜力^［16］^。从化学结构角度分析，以咔唑单元构建的HCP可通过富电子芳香稠环与TCs之间的*π-π*堆积、疏水网络空腔的富集作用以及咔唑氮原子与TCs分子官能团间形成的氢键，实现对四环素类抗生素的多机制协同吸附。基于此，本研究以咔唑为功能单体，FDA为外交联剂，FeCl_3_兼具催化剂与铁源的双重功能，通过傅克烷基化反应首先合成咔唑基超交联聚合物（car-HCP）前驱体，并借助溶剂热法原位负载Fe_3_O_4_纳米粒子，制备了磁性复合材料Fe_3_O_4_/car-HCP，并系统表征了其化学结构、形貌、孔道性质与磁性特征。将其作为MSPE吸附剂，结合高效液相色谱-紫外检测（HPLC-UV）技术，建立了针对环境水样中OTC、TC和DC 3种典型TCs的富集与检测新方法。本研究旨在为环境水体中痕量抗生素的监测提供一种吸附性能优良、分离便捷的分析方法，并为多功能磁性吸附材料的设计合成提供新思路。

## 1 实验部分

### 1.1 仪器、试剂与材料

Nicolet傅里叶红外光谱仪（美国Thermo Scientific公司）；D/max 2500V X射线衍射仪（日本理学公司）；ASAP2020氮气吸脱附测试比表面积分析仪（美国Micromeritics 公司）；FEI TF20场发射冷冻透射电镜（美国Thermo Fisher公司）；Axis UltraDLD X射线光电子能谱仪（英国KRATOS公司）；MERLIN Compact场发射扫描电镜（德国Zeiss公司）；7404型振动样品磁强计（美国Lake Shore公司）；Agilent 1200系列高效液相色谱仪（美国安捷伦公司）。DHG-9146A电热恒温鼓风干燥箱（上海亚荣生化仪器厂）；KQ2200DE数控超声波清洗器（昆山市超声仪器有限公司）；DF-101S集热式恒温加热磁力搅拌器（河南省予华仪器有限公司）；ZKXF电热真空干燥箱（上海树立仪器仪表有限公司）；PHS3C pH计（北京仪电科学仪器股份有限公司）；SHZ-D循环水多用真空泵（河南省予华仪器有限公司）。

咔唑（纯度99.5%）、FeCl_3_（分析纯）、聚乙二醇（PEG，分析纯）、DC（≥98%）、OTC（≥98%）、TC（色谱纯）均购于麦克林生化科技股份有限公司（上海）。FDA（纯度98%）、二氯乙烷（DCE，分析纯）、无水乙酸钠（NaOAc，分析纯）、草酸（OA，纯度99.9%）、氢氧化钠（NaOH，分析纯）均购自科密欧化学试剂有限公司（天津）。乙二醇（EG，分析纯）购于凯通化学试剂有限公司（天津）。分析纯甲醇（MeOH）购于天力化学试剂有限公司（天津）。色谱纯甲醇、乙腈（ACN）购于四友精细化学品有限公司（天津）。

### 1.2 分析条件

Atlantis T3-C18色谱柱（250 mm×4.6 mm，5 μm），流速1.0 mL/min，检测波长为365 nm，流动相为乙酸（0.01 mol/L）（A）和甲醇-乙腈（体积比为1∶1）（B），等度洗脱程序A∶B=63∶37（体积比），进样体积：20 μL。

### 1.3 Fe_3_O_4_/car-HCP复合材料的制备

car-HCP的合成参照文献［^17^］报道的方法，对其中的反应物用量进行了调整。称取咔唑（0.334 g，2 mmol）和FDA（1.06 mL，12 mmol）于单口圆底烧瓶中，加入20 mL DCE（经无水氯化钙干燥）和无水FeCl_3_催化剂（1 g，18 mmol），超声10 min左右使其混合均匀。将混合物于45 ℃磁力搅拌装置中反应5 h后，升温至80 ℃，再继续反应19 h；反应结束后冷却1 h，旋蒸以除去多余溶剂，得到car-HCP。所得浓缩物不经任何纯化处理，直接用于下一步反应。

Fe_3_O_4_/car-HCP复合材料根据文献［^18^，^19^］方法稍作修改制备。准确称取无水乙酸钠（2 g）和PEG（0.6 g）于上述盛有浓缩物的圆底烧瓶中，加入乙二醇（20 mL），超声10 min并在常温下搅拌30 min使其混合均匀。将混合液转移至50 mL反应釜中，在温度200 ℃的高温烘箱中反应12 h。在此溶剂热条件下，体系中的Fe³^+^原位转化为Fe_3_O_4_纳米颗粒，并负载于car-HCP骨架中。用甲醇和蒸馏水交替洗涤产品并用磁铁对所得产品进行分离，通过索氏提取（以甲醇为溶剂，提取8 h）来对产品进一步纯化，然后将产品放入真空干燥箱中，在60 ℃的真空条件下干燥24 h，最后得到黑色粉末Fe_3_O_4_/car-HCP复合材料。制备过程如图1所示。

**图1:**
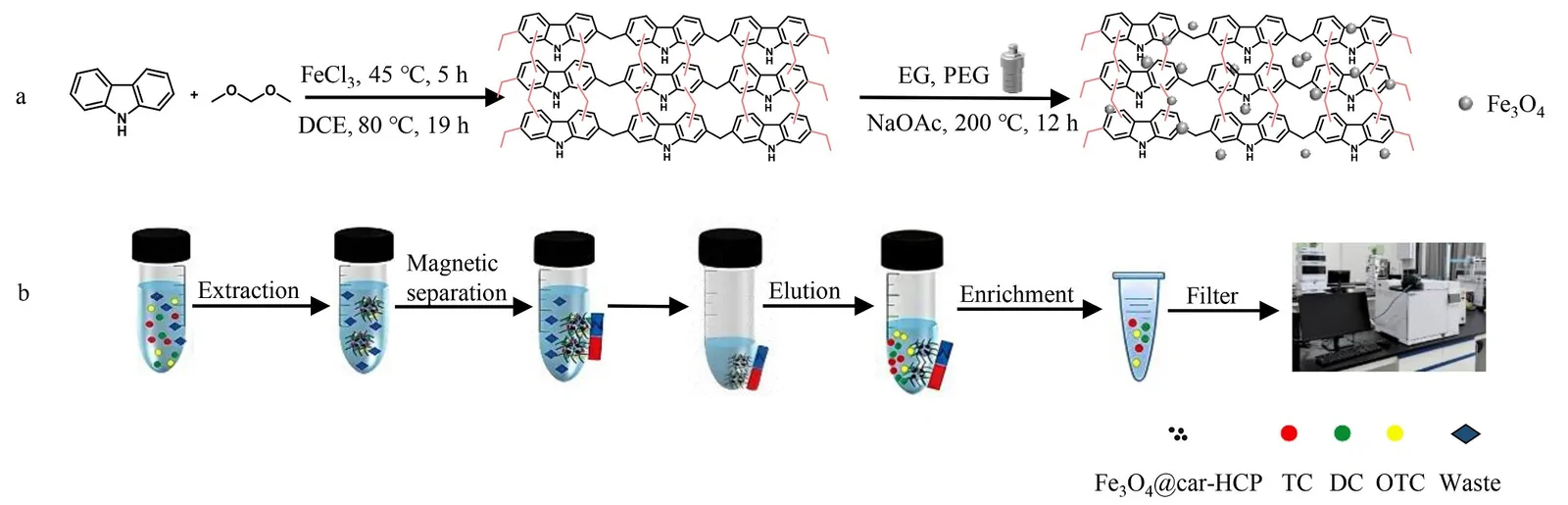
（a）Fe_3_O_4_/car-HCP的合成过程示意图以及（b）从水样中提取TC、DC和OTC的MSPE流程图 DCE： dichloroethane； EG： ethylene glycol； PEG： polyethylene glycol； NaOAc： acetic acid，sodium salt； HCP： hypercrosslinked polymer； TC： tetracycline； DC： doxycycline； OTC： oxytetracycline.

### 1.4 MSPE步骤

取25 mL水样（或不同浓度的TCs标准水溶液），加入一定量的Fe_3_O_4_/car-HCP磁性吸附剂，在30 ℃下进行超声辅助萃取直至吸附达到平衡。随后，利用外加磁体实现固液分离，弃去上清液。向所得磁性吸附剂中加入3 mL洗脱剂，通过超声处理使目标分析物完全脱附。再次施加外磁场，分离吸附剂与洗脱液，收集洗脱液。将洗脱液在温和氮气流下吹干，残留物用1.0 mL流动相重新溶解，经0.22 μm尼龙6有机相滤膜过滤后，进行HPLC-UV检测与定量分析。整个前处理与检测流程见图1。

## 2 结果与讨论

### 2.1 Fe_3_O_4_/car-HCP的表征

采用傅里叶变换红外光谱（FT-IR）对car-HCP及Fe_3_O_4_/car-HCP复合材料的结构进行了表征，结果如图2a所示。对于car-HCP，谱图中位于3 677 cm⁻¹处的吸收峰归属于咔唑单元中吡咯基团的N-H伸缩振动^［20］^；位于1 486、1 602及1 660 cm⁻¹处的峰则对应于咔唑芳香杂环的骨架振动^［20］^。此外，在2 920 cm⁻¹处观察到的明显吸收峰为亚甲基（-CH_2_-）的伸缩振动特征峰，该峰的出现证实了超交联网络的成功构建^［21］^。在Fe_3_O_4_/car-HCP的红外谱图中，除了上述所有特征峰外，在580 cm⁻¹处出现了归属于Fe_3_O_4_中Fe-O键伸缩振动的特征吸收峰^［17］^，证明Fe_3_O_4_纳米粒子已成功负载于car-HCP骨架上，实现了材料的磁功能化。为评估该复合材料的化学稳定性，将Fe_3_O_4_/car-HCP分别置于pH 1和pH 14环境中浸泡15 h，处理后材料的FT-IR谱图与原始谱图相比未发生明显变化，表明其在极端酸碱条件下仍具备良好的结构稳定性。

**图2:**
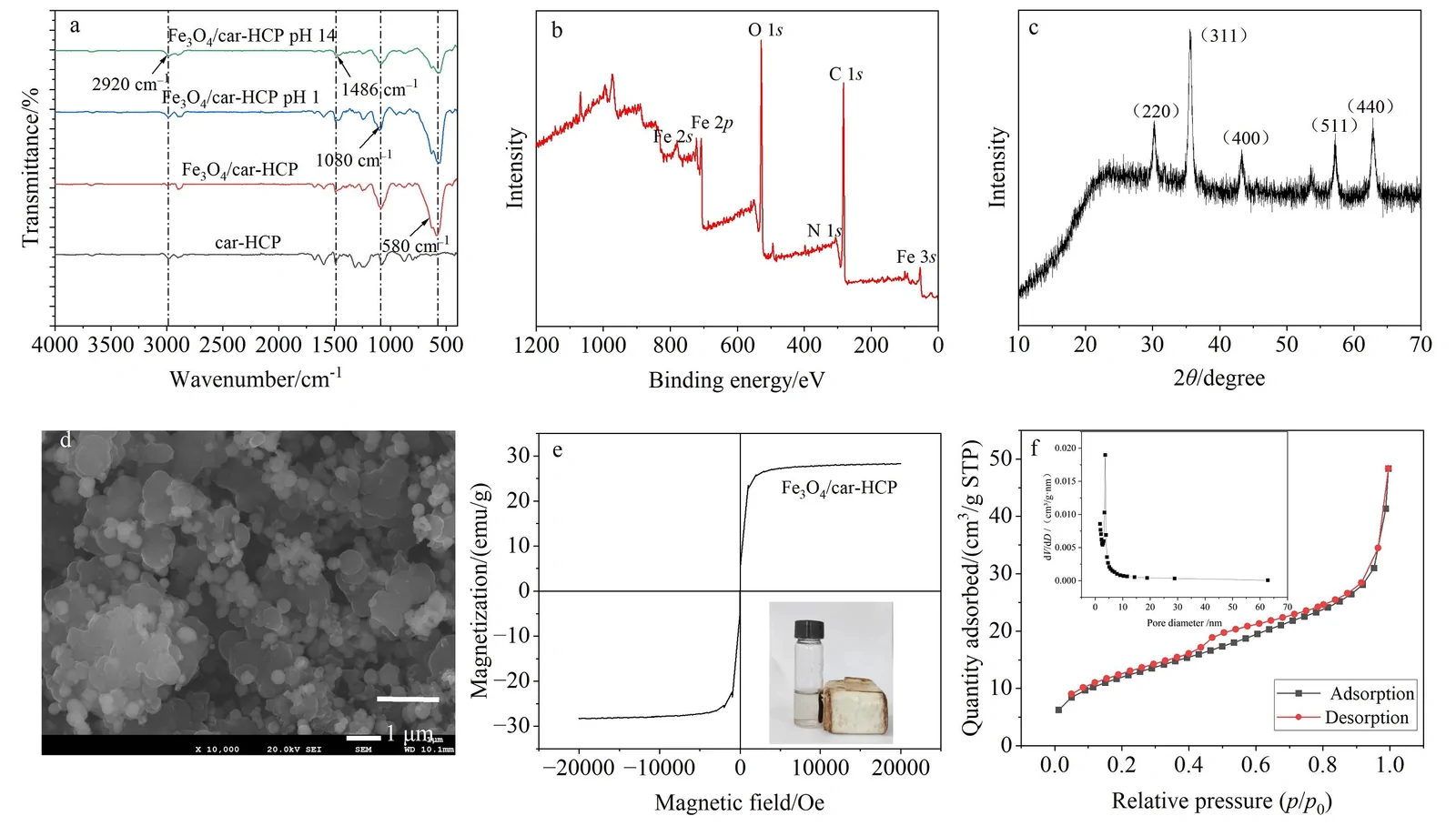
（a）car-HCP和Fe_3_O_4_/car-HCP的红外谱图；Fe**_3_**O**_4_**/car-HCP的（b）XPS全谱分析、（c）XRD图、（d）SEM图、（e）磁化曲线和（f）氮气吸附-脱附等温线 XPS： X-ray photoelectron spectroscopy； XRD： X-ray diffraction； SEM： scanning electron microscope. Fig. 2f： The inset shows the corresponding pore size distribution.

通过X射线光电子能谱（XPS）对Fe_3_O_4_/car-HCP进行元素组成分析，其全谱图（图2b）证实材料主要由C、N、O、Fe 4种元素构成，未见其他元素特征峰，结果与其化学组成一致。

通过X射线衍射（XRD）进一步确定了Fe_3_O_4_的晶相结构，结果如图2c所示。Fe_3_O_4_/car-HCP的XRD图谱在2*θ*为30.1°、35.5°、43.1°、57.0°和62.6°处出现了5个尖锐的衍射峰，分别对应于立方相Fe_3_O_4_（JCPDS card NO. 82-1533）的（220）、（311）、（400）、（511）和（440）晶面，证实了Fe_3_O_4_纳米粒子在car-HCP上的成功负载与磁化。图2d是Fe_3_O_4_/car-HCP材料的扫描电镜图，从中可以看出球形的Fe_3_O_4_磁性纳米粒子，与不规则块状的car-HCP紧密均匀地结合成复合体。

图2e是Fe_3_O_4_/car-HCP的磁化曲线，曲线关于原点对称，表明材料具有超顺磁性，无磁滞、剩磁或矫顽力^［22］^。该材料具有足够的磁响应强度满足分离要求，并且当外部磁场移除时可以快速再分散。N_2_吸附-脱附等温线（图2f）表明Fe_3_O_4_/car-HCP的Langmuir比表面积为183.148 5 m^2^/g，平均孔径为7.1 nm，这有利于吸附过程中的质量传递，为TCs分子提供了有效的扩散通道与丰富的可接触表面。

### 2.2 磁性固相萃取条件优化

为了优化Fe_3_O_4_/car-HCP对TCs的萃取条件和参数，考察了吸附剂用量、萃取时间、萃取温度、洗脱时间、洗脱剂类型等参数对Fe_3_O_4_/car-HCP萃取效果的影响。

吸附剂用量是影响MSPE回收率的关键因素之一，本实验在25 mL质量浓度为100 μg/L的TC、DC、OTC混合标准溶液中，分别考察了5、10、15、20、25、30 mg Fe_3_O_4_/car-HCP吸附剂对TCs回收率的影响。如图3a所示，吸附剂用量在5~20 mg范围内增加时，TC、DC和OTC的回收率显著上升，主要归因于有效吸附位点的增多；超过20 mg后回收率趋于稳定，表明吸附趋于饱和，故确定最佳用量为20 mg。

**图3:**
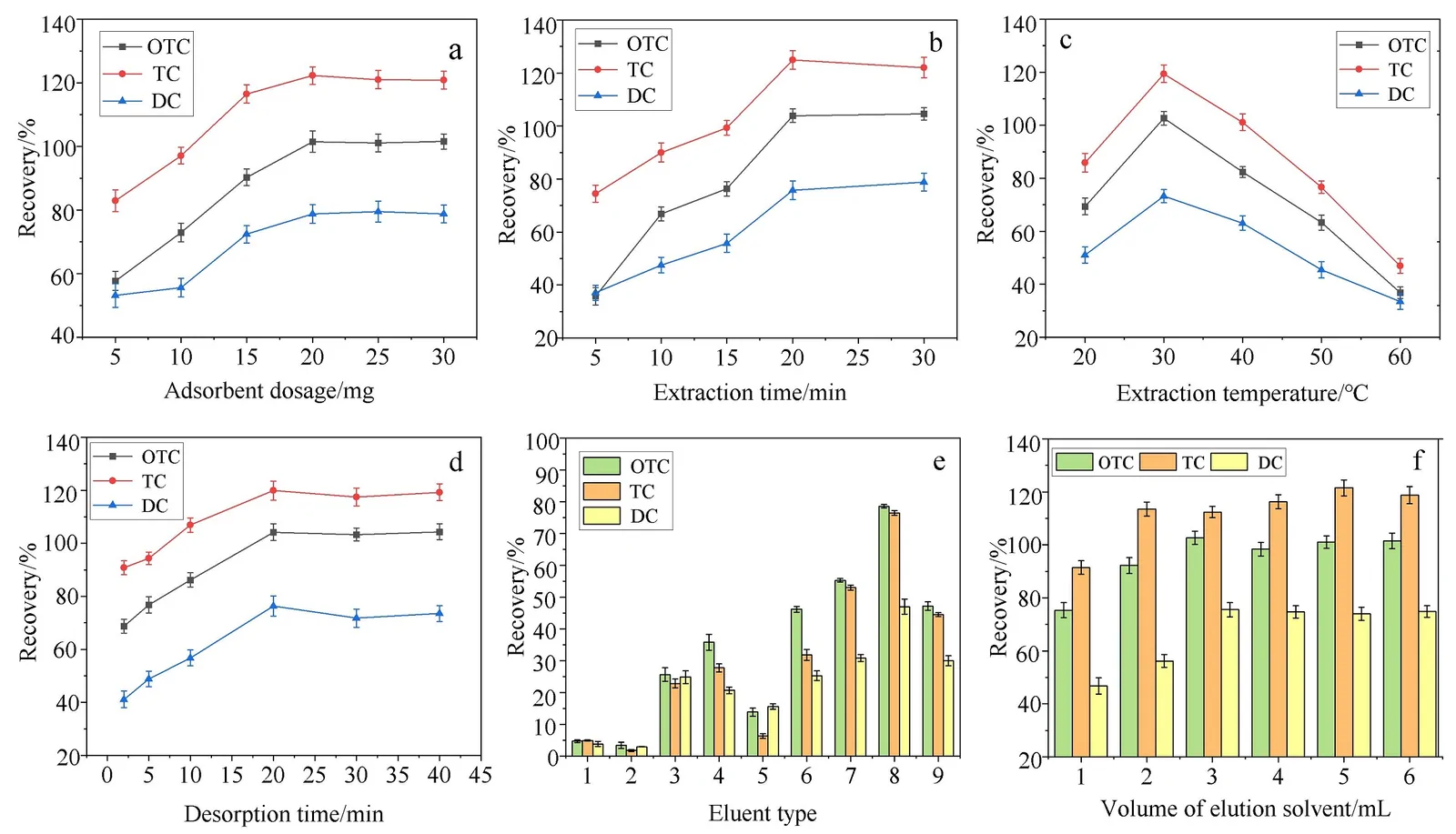
（a）吸附剂用量、（b）萃取时间、（c）萃取温度、（d）洗脱时间、（e）洗脱剂类型、（f）洗脱体积对TCs回收率的影响（*n*=3） For Fig. 3e： 1. MeOH； 2. ACN； 3. MeOH-0.1 mol/L HCl （1∶1，volume ratio）； 4. ACN-0.1 mol/L HCl （1∶1，volume ratio）； 5. MeOH-NH_3_·H_2_O （2∶1，volume ratio）； 6. ACN-NH_3_·H_2_O （2∶1，volume ratio）； 7. MeOH-0.01 mol/L NaOH （2∶1，volume ratio）； 8. ACN-0.01 mol/L NaOH （2∶1，volume ratio）； 9. ACN-0.01 mol/L NaOH （3∶1，volume ratio）. For Fig. 3f，the elution solvent is ACN-0.01 mol/L NaOH （2∶1，volume ratio）.

本实验在5~30 min内优化了萃取时间对MSPE回收率的影响，结果如图3b所示。萃取时间在5~30 min内延长时，3种目标物的回收率总体呈上升趋势，其中OTC和DC的回收率持续提高，而TC的回收率在达到峰值后略有下降，但30 min时仍维持在较高水平。综合考虑萃取效率与实验周期，本研究最终选择30 min为最佳萃取时间。

温度会影响分析物的质量转移率和分配系数，因此考察了20~60 ℃的萃取温度对MSPE回收率的影响。如图3c所示，当温度从20 ℃升高到30 ℃时，回收率显著增加，30 ℃时达到最高，继续升温则回收率下降，因此选择30 ℃为最佳萃取温度。

图3d考察了2~40 min内不同洗脱时间对MSPE回收率的影响。在2~20 min范围内，随着洗脱时间的增加，TC、DC、OTC的回收率不断升高；20 min后回收率保持稳定，故在后续实验中选用的洗脱时间为20 min。

通过改变洗脱剂的种类，优化了洗脱剂对TC、OTC、DC的洗脱效率。为了破坏目标分析物与Fe_3_O_4_/car-HCP之间的疏水作用和*π-π*作用，选择了常用的混合有机溶剂（如甲醇和乙腈各2 mL）。为了增加洗脱剂的亲水性并破坏目标分析物与Fe_3_O_4_/car-HCP间的氢键作用，分别在甲醇和乙腈中加入了一定浓度的稀酸和稀碱作为洗脱剂。结果如图3e所示，纯甲醇或乙腈的洗脱效果较差，采用甲醇或乙腈与稀碱的混合溶液可显著提高回收率。比较了两种稀碱溶液（氨水和NaOH）的洗脱效果，发现NaOH的洗脱效果比氨水更好，因此选择乙腈和0.01 mol/L的NaOH溶液作为洗脱液。其中乙腈-0.01 mol/L NaOH（体积比为2∶1）的洗脱效率最高。

为了考察洗脱剂体积对萃取回收率的影响，对洗脱剂乙腈-0.01 mol/L NaOH（体积比为2∶1）进行了优化。结果如图3f所示，OTC、DC在洗脱液体积为3 mL时回收率达到最高，TC在洗脱液体积为2~5 mL时回收率均超过100%。综合考虑，选择3 mL洗脱剂进行洗脱。

### 2.3 方法学验证

在确定最佳MSPE萃取条件后，对25 mL质量浓度分别为5、10、20、50、100、200 μg/L的TC、OTC和DC混合标准溶液进行富集与分析，并建立了相应的工作曲线（其中DC在5 μg/L浓度点的信噪比（*S/N*）低于10而未纳入标准曲线）。结果表明，3种目标物在各自的范围内线性关系良好：OTC的线性范围为2.83~200 μg/L（决定系数（*R*^2^）=0.998 1），TC为2.77~200 μg/L（*R*^2^=0.992 9），DC为9.93~200 μg/L（*R*^2^=0.997 4）。基于*S/N*=3和*S/N*=10计算方法的检出限（LOD）和定量限（LOQ），OTC、TC和DC的LOD分别为0.83、0.80和2.98 μg/L，LOQ分别为2.83、2.77和9.93 μg/L。各分析物的线性范围下限与其LOQ一致。连续5次平行测定所得各浓度点的相对标准偏差（RSD）为3.1%~10.1%，表明该方法具有良好的重现性。

为评价方法的准确度与适用性，在10 μg/L和100 μg/L两个加标水平下进行了加标回收试验，由实验结果（表1）可知，TC、OTC和DC在不同水样中的加标回收率范围为75.6%~128.5%，所有回收率值均在可接受范围内，表明该方法具有良好的准确度；在10 μg/L和100 μg/L两个加标水平下，3种目标物在不同实际水样中的回收率RSD为1.3%~9.4%，说明方法具有较好的重复性与稳定性。综上所述，本研究建立的MSPE-HPLC方法具有较宽的线性范围、较低的检出限及良好的分析精度，适用于环境水样中痕量四环素类抗生素的定量检测。

**表1:** TCs在自来水、雨水及3种湖水中的加标回收率及精密度（*n*=3）

TCs	Spiked/（μg/L）	Tap water			Rainwater			Liangyuan Lake			Beilong Lake			Xiliu Lake		
		Found/（μg/L）	Rec./%	RSD/%	Found/（μg/L）	Rec./%	RSD/%	Found/（μg/L）	Rec./%	RSD/%	Found/（μg/L）	Rec./%	RSD/%	Found/（μg/L）	Rec./%	RSD/%
OTC	10	8.65	86.5	5.1	8.88	88.8	9.4	9.85	98.5	8.9	12.7	127.0	3.2	9.2	92.2	3.9
	100	110.8	110.8	4.2	108.2	108.2	6.2	117.8	117.8	3.1	86.1	86.1	5.6	96.4	96.4	3.2
TC	10	8.75	87.5	4.7	8.25	82.5	8.7	12.9	128.5	7.3	12.1	121.0	3.2	9.3	93.0	8.1
	100	107.3	107.3	1.3	121.3	121.3	5.4	108.9	108.9	5.5	86.7	86.7	5.6	110.9	110.9	7.6
DC	10	7.76	77.6	3.6	7.65	76.5	6.3	8.3	83.0	2.9	8.15	81.5	7.7	7.86	78.6	2.3
	100	80.1	80.1	5.9	80.4	80.4	4.3	81.5	81.5	4.6	75.6	75.6	7.3	76.9	76.9	6.5

Rec.： recovery.

### 2.4 实际样品的分析测定

采用所建立的基于Fe_3_O_4_/car-HCP的MSPE-HPLC方法，对自来水、雨水及3种湖水（粮苑湖、北龙湖、西流湖）等实际环境水样中OTC、TC和DC进行了检测，结果表明，所有水样中均未检出上述3种抗生素；西流湖水样的色谱图见图4。

**图4:**
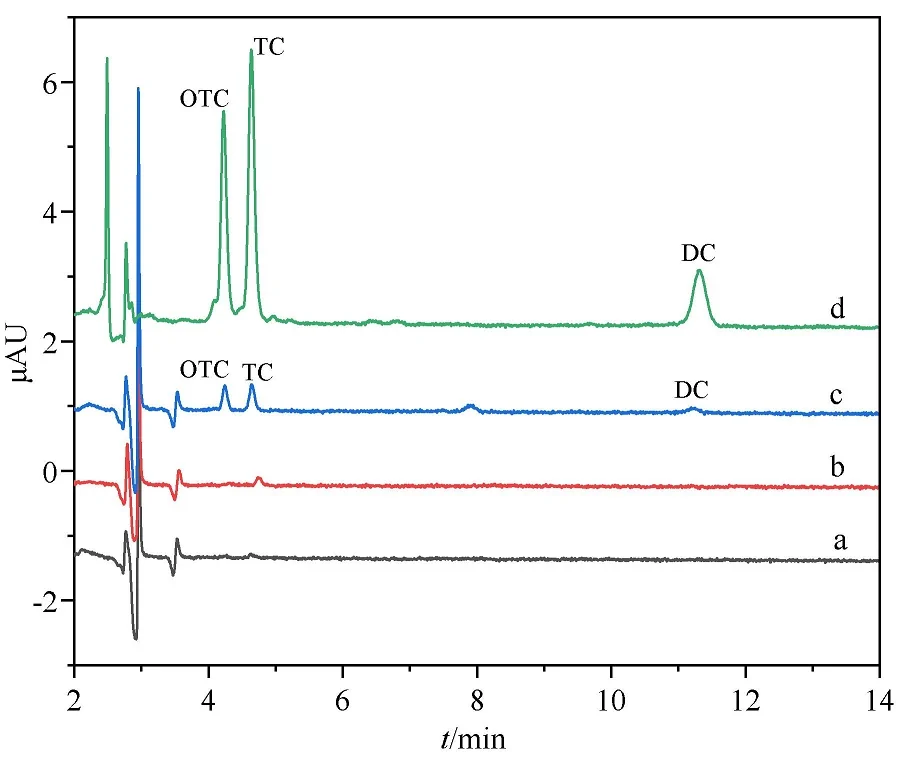
西流湖水样的色谱图 a. blank sample； b. blank sample spiked with 10 μg/L of TCs，before enrichment； c. blank sample spiked with 10 μg/L of TCs，after enrichment； d. blank sample spiked with 100 μg/L of TCs，after enrichment.

### 2.5 Fe_3_O_4_/car-HCP的重复使用性及与其他吸附材料比较

吸附剂的重复使用性是影响其实际应用的重要因素，对于使用过的磁性吸附剂材料Fe_3_O_4_/car-HCP，用乙腈-0.01 mol/L NaOH水溶液（体积比为2∶1）超声洗脱30 min后用蒸馏水洗涤3次，然后用于下一次MSPE萃取程序。从图5可以看出，循环使用5次后，TC、OTC的MSPE回收率依然在80%以上，DC的萃取回收率也维持在60%以上，表明Fe_3_O_4_/car-HCP对不同目标物的重复使用性能存在差异。

**图5:**
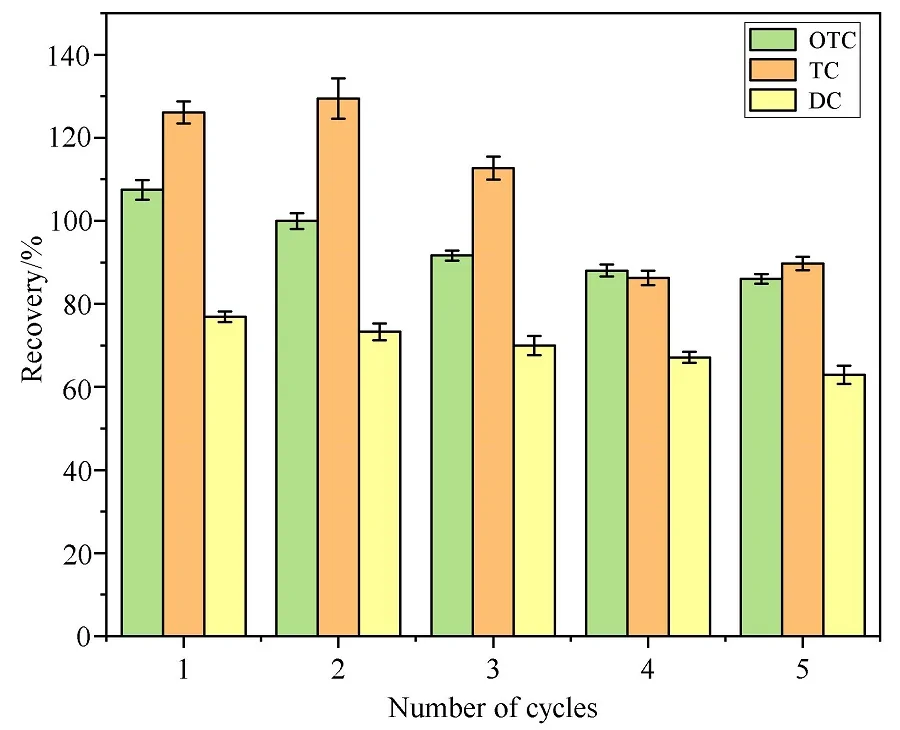
Fe_3_O_4_/car-HCP循环使用5次对TCs回收率的影响（*n*=3）

从实验操作的简便性、资源消耗（吸附剂用量、分析时间）及方法学性能（线性范围、检出限、回收率）等多个维度，将本方法与已报道的TCs萃取方法进行了系统比较（见表2）。本方法采用MSPE与自制磁性HCP吸附剂相结合，通过外加磁场实现快速固液分离，避免了传统SPE（如ZIF-8、MIPs、Oasis HLB等）所需的过柱或离心步骤，大幅提升了前处理的便捷性；同时吸附剂用量少，分析时间短，与多数磁性方法（如M-G/CNTs、mag-AC）相当，却显著优于传统SPE（如ZIF-8、MIPs、Oasis HLB和Oasis PRiME HLB）方法，在材料经济性和前处理效率上优势明显。在检测手段与成本方面，本方法采用普及度更高的UV检测器，避免了质谱联用（如SCAU-1/SNW-1、Oasis HLB、Fe_3_O_4_@ZIF-8等方法所需的MS/MS）带来的高昂仪器成本，更适用于常规实验室的快速分析。在方法学性能方面，本方法的LOD更低，优于ZIF-8、PVP/SWCNT-polyHIPE及M-G/CNTs等材料，虽不及质谱联用方法的痕量水平，但在非质谱检测方法中处于较优水平；线性范围与多数方法（如MIPs、mag-AC）相当或更宽。综上，本方法兼具操作简便与成本较低的特点，同时实现了较低的检出限与良好的回收率，适用于常规实验室中痕量分析的前处理环节。

**表2:** 本研究与其他研究在方法、吸附剂用量、线性范围、LOD以及回收率方面的对比

Sorbent	Method	Mass of sorbent/mg	Analysis time/min	Linear range^*^/ （μg/L）	LOD/（μg/L）	Recovery/%	Ref.
ZIF-8	SPE/HPLC-PAD	390	~55	5-1000	1.50-8.00	70.3-107.4	［^23^］
MIPs	SPE/HPLC-UV	400	~75	1-200	0.08-1.02	83.2-111.0	［^24^］
SCAU-1 and SNW-1	SPE/UPLC-MS/MS	60	~35	1-500	0.30-0.60	88.4-93.4	［^25^］
PVP/SWCNT-polyHIPE	SPE/HPLC-FLD	60	~80	20-800	1.00–2.50	85.0-101.7	［^26^］
Oasis HLB	SPE/UPLC-MS/MS	500	~170	0-50	3.18×10^-6^-1.0×10^-4^	80.4-104.2	［^27^］
Fe_3_O_4_@ZIF-8	SPE/UHPLC-MS/MS	120	~50	0.005-0.05	（1.2-1.4）×10^-4^	78.3-96.7	［^28^］
M-G/CNTs	MDSPE/HPLC-FLD	20	~30	20-800	3.60	95.5-112.5	［^29^］
mag-AC	MSPE/HPLC-MS/MS	20	~40	0.5-100	0.10-0.95	91.0-102.0	［^30^］
Oasis PRiME HLB	SPE/UPLC-MS/MS	200	~65	1-500	（1.0-1.5）×10^-4^	62.6-119.0	［^31^］
Fe_3_O_4_/car-HCP	MSPE/HPLC-UV	20	~65	2.77-200	0.80-2.98	75.6-128.5	this work

ZIF-8： Zn（MIM）_2_ （MIM=2-methylimidazolate）； MIPs： molecularly imprinted polymers； SCAU-1： a novel triazine-based covalent organic framework； SNW-1： a melamine-based porous covalent organic framework； PVP/SWCNT-polyHIPE： polyvinylpyrrolidone/single-walled carbon nanotube incorporated polymerized high internal phase emulsion； Oasis HLB： hydrophilic-lipophilic balanced polymer solid phase extraction column，specification is 6 mL/500 mg； Fe_3_O_4_@ZIF-8： zeolitic imidazolate frameworks with Fe_3_O_4_ particles； M-G/CNTs： magnetic graphene/carbon nanotube composite materials； mag-AC： magnetic activated carbon. * The linear ranges refer to the lowest and highest values of the linear ranges for all TCs.

## 3 结论

本实验以咔唑为单体、FDA为交联剂、无水FeCl_3_为催化剂，通过傅克烷基化反应合成了car-HCP；在此基础上，进一步利用反应体系中的FeCl_3_作为铁源，通过溶剂热反应实现原位磁化，成功制备了磁性多孔复合材料Fe_3_O_4_/car-HCP。该材料具备制备简便、原料易得、结构稳定等优点，将其用作MSPE吸附剂，结合HPLC-UV，建立了一种用于环境水样中四环素类抗生素OTC、TC和DC的高效分析方法。方法优化后表现出良好的线性范围、较低的检出限，对实际样品的加标回收率为75.6%~128.5%，满足痕量分析要求；且材料可重复使用，在酸碱环境中保持稳定。本研究为环境中痕量四环素类抗生素的检测提供了一种简便、灵敏的前处理方法，同时也为兼具磁分离功能与吸附性能的多孔复合材料设计提供了可行的合成策略。
